# Supplementary material for: Unveiling Cryptosporidium parvum sporozoite-derived extracellular vesicles: profiling, origin, and protein composition
Source: Front Cell Infect Microbiol. 2024 Apr 10;14:1367359. doi: 10.3389/fcimb.2024.1367359 (PMC11039866; doi:10.3389/fcimb.2024.1367359)
Supplement: Supplementary file 4 [file DataSheet_1.pdf]

# NANOSIGHT

# 10K_1a10 2024-02-27 14-03-58

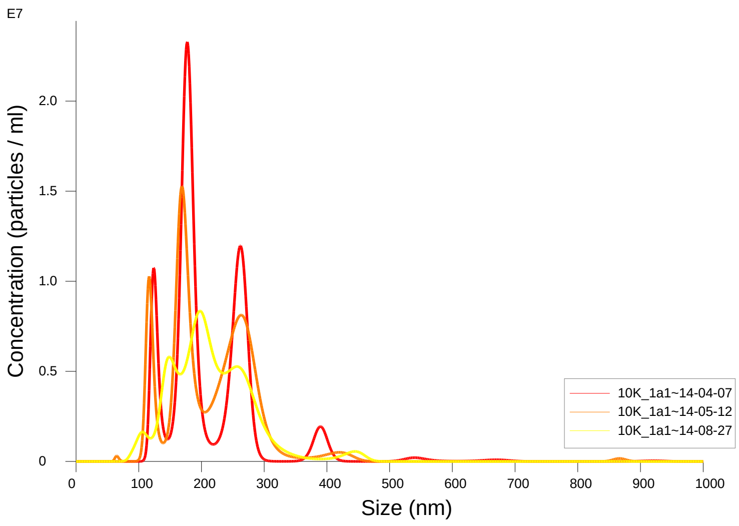


FTLA Concentration / Size graph for Experiment:
10K_1a10 2024-02-27 14-03-58

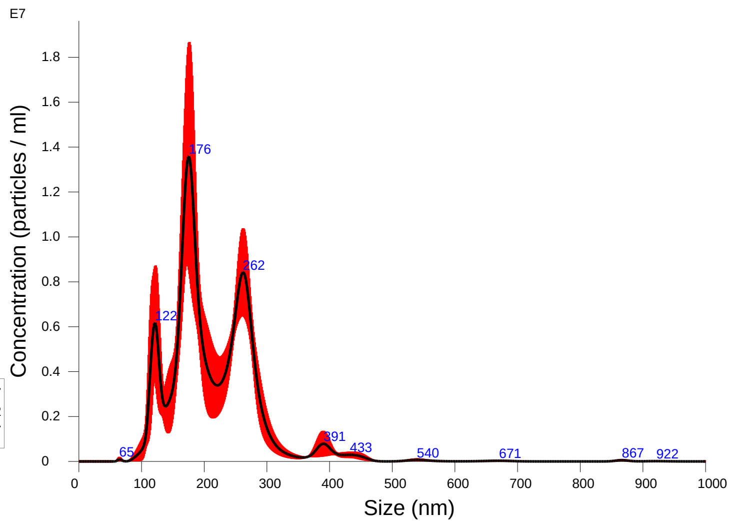


Averaged FTLA Concentration / Size for Experiment:
10K_1a10 2024-02-27 14-03-58
Error bars indicate + / -1 standard error of the mean

## Included Files

10K_1a10 2024-02-27 14-04-07
10K_1a10 2024-02-27 14-05-12
10K_1a10 2024-02-27 14-08-27

## Details

| | |
|---|---|
| NTA Version: | NTA 3.4 Build 3.4.4 |
| Script Used: | SOP Standard Measurement 02-03-42PM 27~ |
| Time Captured: | 14:03:58  27/02/2024 |
| Operator: | |
| Pre-treatment: | |
| Sample Name: | |
| Diluent: | |
| Remarks: | |

## Capture Settings

| | |
|---|---|
| Camera Type: | sCMOS |
| Laser Type: | Blue488 |
| Camera Level: | 14 |
| Slider Shutter: | 1259 |
| Slider Gain: | 245 |
| FPS | 25.0 |
| Number of Frames: | 1498 |
| Temperature: | 22.8 - 23.1 $^{o}$C |
| Viscosity: | (Water) 0.929 - 0.936 cP |
| Dilution factor: | 1 x 10e1 |
| Syringe Pump Speed: | 30 |

## Analysis Settings

| | |
|---|---|
| Detect Threshold: | 5 |
| Blur Size: | Auto |
| Max Jump Distance: | Auto: 11.0 - 11.8 pix |

## Results

Stats: Merged Data

| | |
|---|---|
| Mean: | 214.0 nm |
| Mode: | 175.2 nm |
| SD: | 74.6 nm |
| D10: | 130.8 nm |
| D50: | 195.8 nm |
| D90: | 283.8 nm |

Stats: Mean +/- Standard Error

| | |
|---|---|
| Mean: | 214.1 +/- 1.0 nm |
| Mode: | 181.3 +/- 8.6 nm |
| SD: | 73.9 +/- 4.5 nm |
| D10: | 131.2 +/- 5.8 nm |
| D50: | 197.9 +/- 6.7 nm |
| D90: | 284.0 +/- 4.7 nm |
| Concentration (Upgrade): | 1.16e+09 +/- 6.14e+07 particles/ml |
| | 20.2 +/- 1.1 particles/frame |
| | 23.5 +/- 1.3 centres/frame |

# NANOSIGHT

## 10K_1a10 2024-02-27 14-03-58

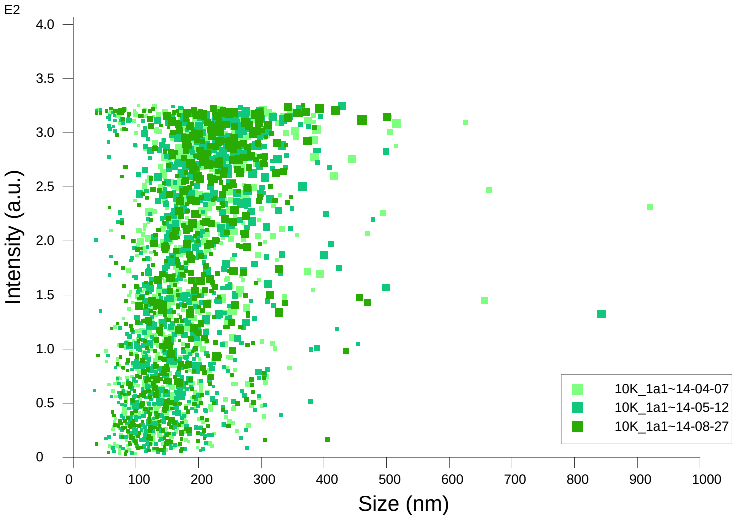


Intensity / Size graph for Experiment:
10K_1a10 2024-02-27 14-03-58

**Script Used: (Full Text):**

SOP Standard Measurement 02-03-42PM 27Feb2024.txt
