# Supplementary material for: Unveiling Cryptosporidium parvum sporozoite-derived extracellular vesicles: profiling, origin, and protein composition
Source: Front Cell Infect Microbiol. 2024 Apr 10;14:1367359. doi: 10.3389/fcimb.2024.1367359 (PMC11039866; doi:10.3389/fcimb.2024.1367359)
Supplement: Supplementary file 5 [file DataSheet_2.pdf]

NANOSIGHT

100k_1a10 2024-02-27 14-20-33

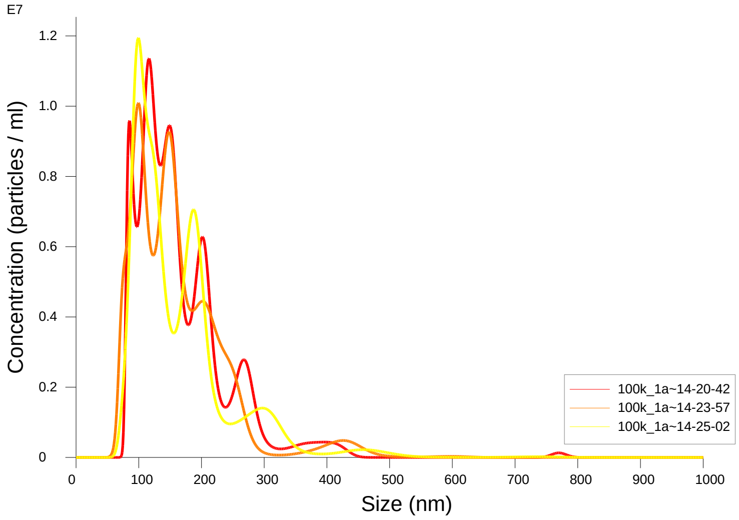

FTLA Concentration / Size graph for Experiment:
100k_1a10 2024-02-27 14-20-33

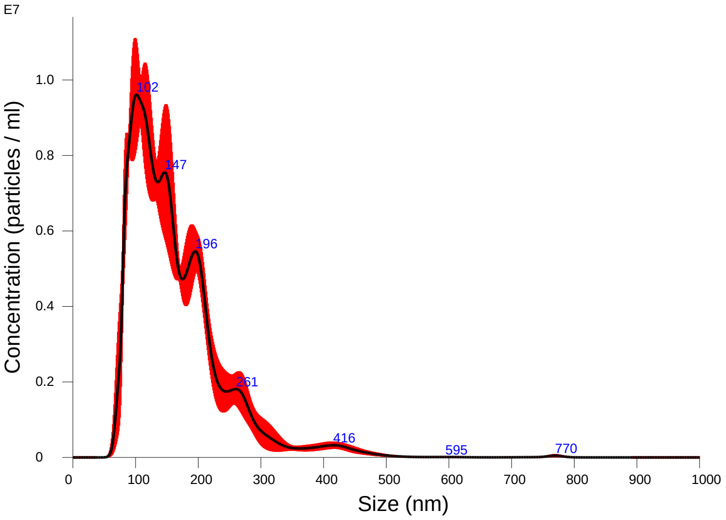

Averaged FTLA Concentration / Size for Experiment:
100k_1a10 2024-02-27 14-20-33
Error bars indicate + / -1 standard error of the mean

Included Files

100k_1a10 2024-02-27 14-20-42
100k_1a10 2024-02-27 14-23-57
100k_1a10 2024-02-27 14-25-02

Details

NTA Version:	NTA 3.4 Build 3.4.4
Script Used:	SOP Standard Measurement 02-19-41PM 27~
Time Captured:	14:20:33 27/02/2024
Operator:	
Pre-treatment:	
Sample Name:	
Diluent:	
Remarks:	

Capture Settings

Camera Type:	sCMOS
Laser Type:	Blue488
Camera Level:	15
Slider Shutter:	1206
Slider Gain:	245
FPS	25.0
Number of Frames:	1498
Temperature:	23.2 - 23.4 ^{o}C
Viscosity:	(Water) 0.922 - 0.926 cP
Dilution factor:	1 x 10e1
Syringe Pump Speed:	30

Analysis Settings

Detect Threshold:	5
Blur Size:	Auto
Max Jump Distance:	Auto: 12.2 - 13.2 pix

Results

Stats: Merged Data

Mean:	163.8 nm
Mode:	101.5 nm
SD:	77.3 nm
D10:	91.0 nm
D50:	145.7 nm
D90:	256.9 nm

Stats: Mean +/- Standard Error

Mean:	163.8 +/- 1.4 nm
Mode:	105.0 +/- 5.6 nm
SD:	77.4 +/- 1.7 nm
D10:	91.0 +/- 1.7 nm
D50:	144.7 +/- 2.5 nm
D90:	260.2 +/- 9.1 nm
Concentration (Upgrade):	1.16e+09 +/- 3.74e+07 particles/ml
	19.9 +/- 0.6 particles/frame
	22.7 +/- 0.6 centres/frame

100k_1a10 2024-02-27 14-20-33

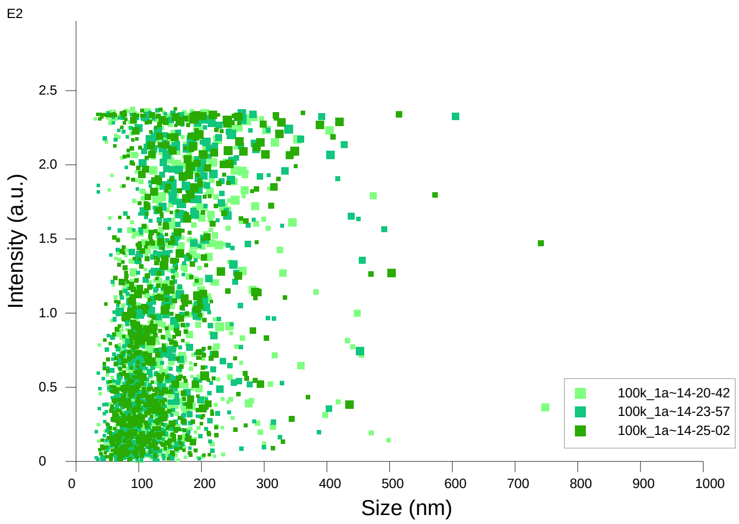

Intensity / Size graph for Experiment:
100k_1a10 2024-02-27 14-20-33

Script Used: (Full Text):

SOP Standard Measurement 02-19-41PM 27Feb2024.txt
