# Supplementary figures and images for: Unveiling Cryptosporidium parvum sporozoite-derived extracellular vesicles: profiling, origin, and protein composition
Source: Front Cell Infect Microbiol. 2024 Apr 10;14:1367359. doi: 10.3389/fcimb.2024.1367359 (PMC11039866; doi:10.3389/fcimb.2024.1367359)

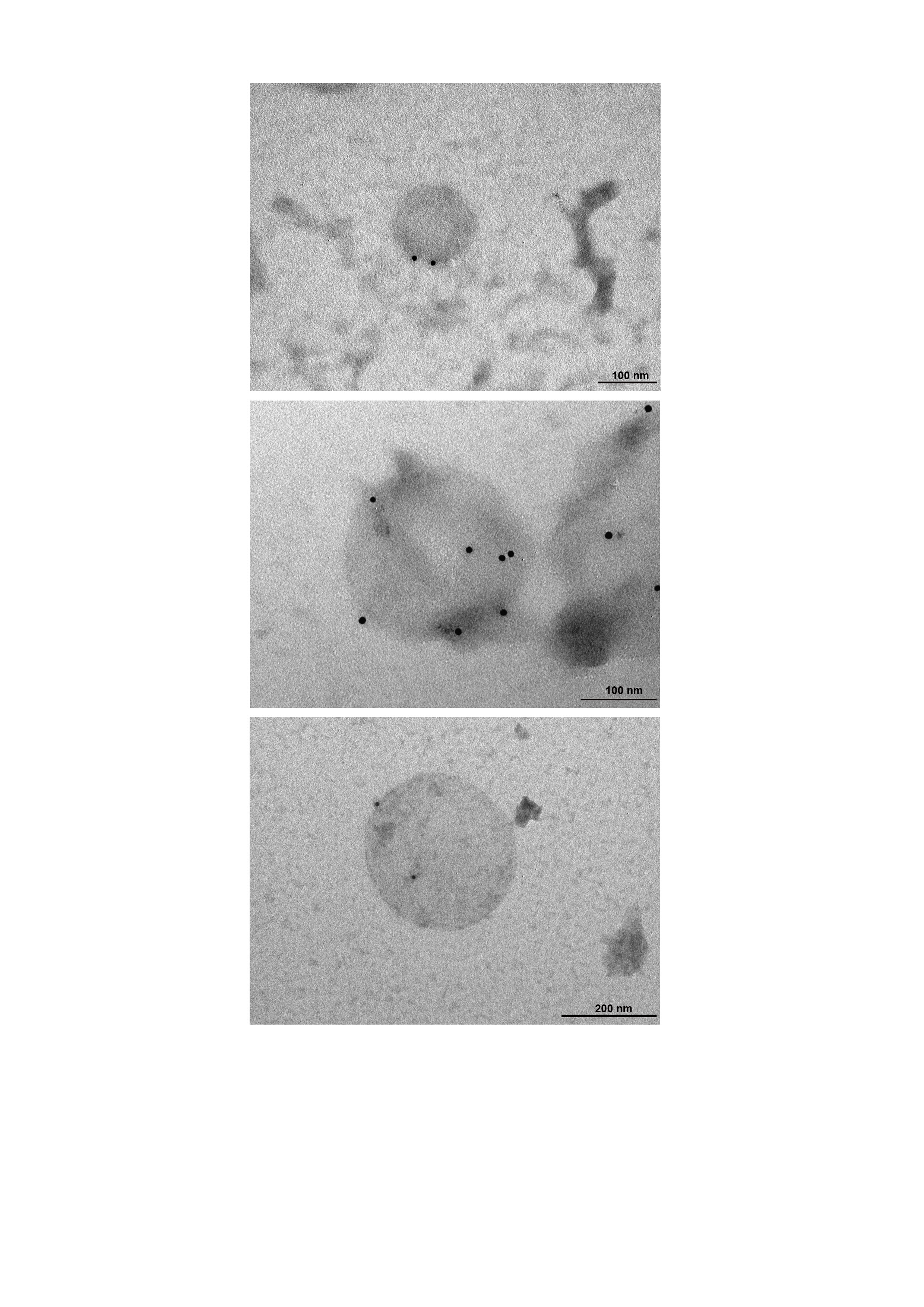

Supplement: Supplementary file 6 [file Image_1.jpeg]

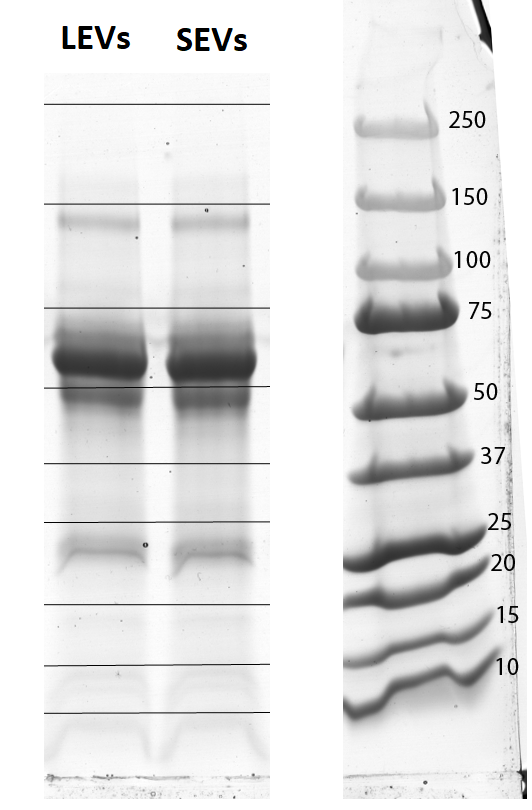

Supplement: Supplementary file 7 [file Image_2.tif]

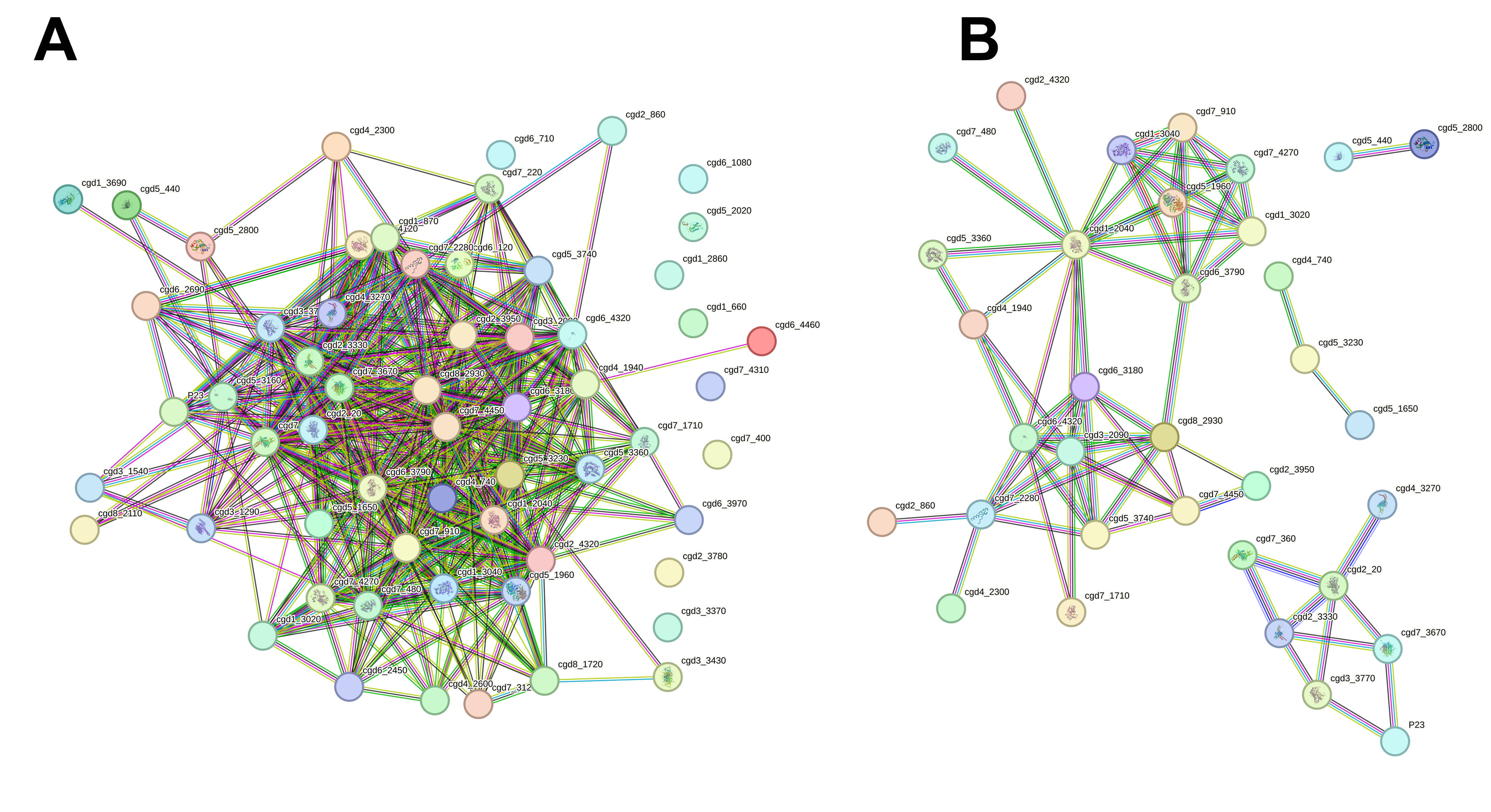

Supplement: Supplementary file 8 [file Image_3.jpeg]
